# The association between thyroid dysfunction, autoimmune thyroid disease, and rheumatoid arthritis disease severity

**DOI:** 10.1186/s12902-023-01473-5

**Published:** 2023-10-05

**Authors:** Mohammad Amin Yazdanifar, Mahsa Bagherzadeh-Fard, Mohammad Amin Habibi, Mostafa Vahedian, Mohammad Bagherzadeh, Maryam Masoumi

**Affiliations:** 1 Qom University of Medical Sciences, Qom, Iran; 2 Research Center for Environmental Pollutants, Qom University of Medical Sciences, Qom, Iran

**Keywords:** Rheumatoid arthritis, Thyroid dysfunction, Anti-thyroid autoantibody, Autoimmune thyroid disease, Disease activity, Disease severity

## Abstract

**Background:**

Rheumatoid Arthritis (RA) and autoimmune thyroid disease (AITD) are the two most prevalent coexisting autoimmune diseases due to their similar pathogenesis. Considering the potential effect of AITD on the severity of RA disease, this study aimed to determine the association between thyroid dysfunction, anti-thyroid peroxidase (anti-TPO) positivity, AITD, and RA disease severity in the Iranian population.

**Methods:**

Three hundred and fifty RA patients who presented to Shahid Beheshti tertiary care center, Qom, Iran, were included in this cross-sectional study. The data were collected through the patient’s medical records, interviews, physical examinations, and laboratory tests. The RA disease activity score in 28 joints for RA with erythrocyte sedimentation rate (DAS-28-ESR) was used to divide patients into three subgroups, remission (DAS-28-ESR ⩽ 2.6), mild-to-moderate (2.6 < DAS-28-ESR ⩽ 5.1), and severe disease activity (DAS-28-ESR > 5.1).

**Results:**

Using the aforementioned method, 111, 96, and 138 patients were put into remission, mild-to-moderate, and severe disease activity groups, respectively. Anti-TPO antibody positivity rate was 2.93 times more prevalent among patients with severe disease compared to the remission subgroup (OR: 2.93; P-value < 0.001). Patients suffering from a more severe disease were almost 2.7 times more probable to have AITD (OR = 2.71; P-value < 0.001) and they were 82% more likely to have thyroid dysfunction compared to patients in remission (OR = 1.82; P-value = 0.006).

**Conclusions:**

It was demonstrated that thyroid dysfunction, anti-TPO antibody positivity, and AITD were significantly more common among RA patients with more severe disease activity.

## Background

Rheumatoid arthritis (RA) is a chronic systemic autoimmune disease that damages small symmetrical joints. Pathogenesis of the disease is complex and consists of environmental stimulations and the immune system malfunction in genetically susceptible individuals [1]. Although the hallmark of RA is the articular involvement, it is considered a systemic disease with a multitude of extra-articular involvements [2], Such as subcutaneous nodules, interstitial lung disease, neuropathy, glomerulonephritis, ischemic heart diseases, etc., primarily observed in longstanding patients [3].

Autoimmune thyroid disease (AITD), primarily Graves’ disease and Hashimoto’s thyroiditis, are the leading causes of goiters in non-iodine deficient regions with a prevalence of up to 5% of the general population. Various autoantibodies are produced in AITD, each targeting specific points in thyroid hormone synthesis process [4]. AITDs and other systematic autoimmune diseases such as Systemic Lupus Erythematous, Sjögren’s syndrome, Systemic sclerosis, and RA frequently coexist due to apparent pathogenesis [5].

Thyroid dysfunction also seems to be more prevalent in many autoimmune diseases, including RA [6]. Previous studies within different demographics and geographic locations have demonstrated various rates of AITD prevalence among RA patients [7]. Although the mechanism of AITDs and RA affecting each other is not yet well defined, several studies have proposed autoimmunity as one of the leading causes of their coexistence and influence on each other [4]. Considering the potential impact of thyroid abnormality on the severity of RA disease, and few studies taking racial diversity into account, it is imperative for further studies to be conducted. Therefore, this study has aimed to investigate the association between RA disease severity and thyroid dysfunction, AITD, and anti-thyroid peroxidase (anti-TPO) positivity in the Iranian population.

## Materials and methods

### Study design and participants

In this observational, cross-sectional study, 350 RA patients registered at Shahid Beheshti tertiary care center, Qom, Iran, from April 2021 to March 2022, were included consecutively.

Patients were diagnosed utilizing the American College of Rheumatology and the European League Against Rheumatism (ACR/EULAR) 2010 criteria for RA [8]. All patients were older than 18, and none suffered from concurrent diabetes mellitus, chronic renal or liver disease, malignancy, current infection, and collagen vascular disease except RA. In addition, patients receiving medications with the potential to interfere with thyroid test and possibly cause false positive or negative results (e.g., dopamine agonists, interferon-α, anticonvulsant drugs, amiodarone, lithium, and somatostatin analogs) were excluded from the study. Pregnant women were also excluded.

Before enrollment, all patients gave informed consent, according to the Declaration of Helsinki, and the protocol was approved by the ethics committee residing at the medical university of Qom (7/23/2021; IR.MUQ.REC.1400.042).

### Clinical assessments

The clinical data were obtained through the patient’s medical record, interview, and physical examination. The data included Patients’ demographic features, prior history of thyroid function disease, duration of RA and thyroid disease, current medications, and the number of tender and swollen joints.

Laboratory tests including rheumatoid factor (RF), erythrocyte sedimentation rate (ESR) using the Westergren method, C-reactive protein (CRP) titer based on Biotec method, mutated citrullinated vimentin autoantibodies (anti-MCV) and anti-cyclic-citrullinated peptide antibodies (anti-CCP) based on an enzyme-linked immunosorbent assay (ELISA) method, Thyroid function tests based on chemiluminescent immunoassay (CLIA) method, and anti-TPO antibody using radioimmunoassay were checked. Anti-CCP and anti-MCV values were considered positive for serum levels of more than 5 U/ml and 20 U/ml, respectively. Thyroid function tests included thyroid stimulating hormone (TSH), free thyroxin (FT4), and free triiodothyronine (FT3) were normal in the range of 0.3–3.6 mIU/L, 0.7–1.8 ng/dl, 2.57–4.43 pg/mL, respectively. Anti-TPO antibody was considered positive for serum levels of more than 50 IU/mL.

Thyroid dysfunction is defined as abnormal levels of TSH with or without abnormal FT4 or FT3 [9]. AITD is diagnosed by endocrinologist as current or prior coexistence of thyroid dysfunction with anti-TPO antibody positivity. Thyroid ultrasound was also performed in cases with suspicious findings in the history of physical examinations [7, 10].

The disease activity score in 28 Joints for RA with ESR (DAS-28-ESR), which is a tool for measuring the severity of the disease, was calculated with the following formula: [11]

DAS-28-ESR = 0.56 × √ (TJC-28) + 0.28 × √ (SJC-28) + 0.014 × VAS + 0.70 × ln (ESR).

Where TJC and SJC stand for the tender joint count and swollen joint count, respectively, and the visual analog scale (VAS) represents the patient’s global health assessment of disease activity and his/her overall health is scored subjectively ranging from 0 as the healthiest to 100 as least healthy.

DAS-28-ESR values were categorized as follows:

- Remission: DAS-28-ESR ⩽ 2.6.
- mild disease activity: 2.6 < DAS-28-ESR ⩽ 3.2.
- Moderate disease activity: 3.2 < DAS-28-ESR ⩽ 5.1.
- severe disease activity: DAS-28-ESR > 5.1.

### Statistical analyses

Quantitate and Qualitative variables were described as mean ± standard deviation (SD) and frequency (percentage), respectively. Kolmogorov-Smirnov test, Q–Q, and P-P plots were used to assess the normality of quantitative variables, in which age and FT3 were normal, and all others were not normal. One-way ANOVA test was recruited for normally distributed data and degrees of freedom, the F value (F) was reported. Additionally, Kruskal–Wallis test was recruited for non-normally distributed data and Kruskal-Wallis H (H) was also reported. The Chi-square test and ordinal regression were used to compare and analyze categorical variables between different types of disease activity; moreover, the 95% confidence interval (CI) was reported. Bivariant correlation analysis was used to determine the correlation between quantitative disease activity parameters and thyroid dysfunction test levels. The deletion method was applied where participants had a missing field which was less than 1%. To demonstrate independent factors associated with disease activity, ordinal regression was used. The data analysis was conducted using IBM SPSS Statistics version 26, and the significance of all tests was considered if the P-value < 0.05.

## Results

Out of 350 patients with RA disease, 345 patients were included in this study and 5 patients were excluded due to the lack of sufficient data to calculate DAS-28-ESR. Based on DAS-28-ESR values, 111, 10, 86, and 138 patients were categorized in remission, mild, moderate, and severe phases of disease activity, respectively. Afterwards, patients were sorted into three subsequent groups of 111 patients in remission, 96 patients with mild-to-moderate, and 138 patients with severe disease activity.

The demographic feature and laboratory findings of all patients are presented in Table 1. Among the 112 patients suffering from thyroid dysfunction, 73 individuals had been already diagnosed with thyroid dysfunction prior to the study which all except for two had overt hypothyroidism, but 39 patients were considered new cases and were diagnosed during the study. ESR, CRP, anti-CCP, and anti-MCV were positive in 11 (9.9%), 14 (12.7%), 60 (54.1%), and 39 (35.5%) patients in remission, 45 (46.9%), 54 (56.3%), 55 (57.9%), and 46 (51.7%) patients with mild-to-moderate disease activity, and 106 (76.8%), 102 (73.9%), 91 (65.9%), and 98 (72.1%) patients with severe disease activity, respectively. As it is shown in Table 1, there was a statistically significant difference between remission, mild-to-moderate, and severe groups for FT3 [F (2, 340) = 35.49, P-value < 0.001] and anti-TPO (H = 15.74, P-value < 0.001).

Anti-TPO antibody was positive in 18 (16.2%) patients in remission, 25 (26.9%) patients with mild-to-moderate disease activity, and 58 (43.6%) patients with severe disease activity. Among patients with severe disease activity, it was 2.93 times more probable to detect anti-TPO positive value than the patients in remission (OR = 2.93, 95% CI: 1.86–4.62; P-value < 0.001). The number of patients suffering from thyroid dysfunction and AITD were 27 (24.3%) and 13 (11.7%) in remission, 27 (28.1%), and 10 (10.4%) mild-to-moderate disease activity, and 55 (40.1%) and 37 (28.0%) with severe disease activity, respectively. Hence, the patients with higher disease activity were 82% more likely to have thyroid dysfunction (OR = 1.82, 95% CI: 1.19–2.77; P-value = 0.006) and 2.7 times more probable to have AITD than the remission patients (OR = 2.71, 95% CI = 1.57–4.62; P-value < 0.001).

DAS-28-ESR level was positively correlated with anti-TPO (r = 0.192; P-value < 0.001) and FT3 (r = 0.388; P-value < 0.001), but there was no significant correlation with TSH nor FT4. The dose of Methotrexate and Hydroxychloroquine was only correlated with FT3 (r = 0.371; p-value < 0.001 and r = 0.121; p-value = 0.024, respectively). Yet, Prednisolone dosage was positively correlated with FT3 (r = 0.404; p-value < 0.001) and anti-TPO (r = 0.236; p-value < 0.001) levels but did not have any significant correlation with FT4 and TSH levels.

The parameters that may impact the disease severity in the presence of other accompanying factors are shown in Table 2 to indicate independent predictors affecting RA disease severity. As it is demonstrated higher levels of thyroid function tests, anti-TPO, and female gender are independent predictors which are significantly associated with more severe RA disease activity.

**Table 1:** Parameters of RA patients in three groups of remission, mild-to-moderate, and severe disease activity using ANOVA, chi-square, and Kruskal-Wallis test

Parameters	Remission (n = 111) mean ± SD or n (%)	Mild-to-moderate disease (n = 96) mean ± SD or n (%)	severe disease (n = 138) mean ± SD or n (%)	P-value
Age (year)	52.3 ± 11.5	51.1 ± 12.3	53.5 ± 12.9	0.345
Female	83 (74.8%)	74 (77.1%)	114 (82.6%)	0.300
Duration of RA disease (year)	7.8 ± 8.3	8.8 ± 9.7	6.7 ± 6.6	0.155
Duration of thyroid disease (year)	2.5 ± 7.1	1.4 ± 5.8	3.2 ± 7.0	0.117
Tender joint count	0.1 ± 0.3	3.6 ± 2.7	14.1 ± 6.9	< 0.001
Swollen joint count	0.0 ± 0.2	3.4 ± 2.8	11.5 ± 6.4	< 0.001
VAS	4.7 ± 8.9	49.5 ± 31.7	90.6 ± 17.4	< 0.001
DAS-28-ESR	1.6 ± 0.5	4.1 ± 0.7	6.6 ± 0.9	< 0.001
**Laboratory findings**				
ESR (mm/h)	10.9 ± 6.0	21.8 ± 15.6	40.9 ± 24.0	< 0.001
CRP (mg/dl)	3.2 ± 2.6	11.6 ± 12.8	24.5 ± 25.1	< 0.001
TSH (mIU/L)	2.3 ± 1.6	2.8 ± 2.2	3.4 ± 3.9	0.154
FT3 (pg/mL)	3.0 ± 1.0	3.8 ± 1.7	4.7 ± 1.7	< 0.001
FT4 (ng/dl)	1.5 ± 1.0	1.9 ± 2.3	1.9 ± 2.7	0.219
Anti-TPO (IU/ml)	64.1 ± 138.8	82.8 ± 154.9	146.7 ± 192.0	< 0.001
Anti-CCP (U/ml)	84.5 ± 129.7	87.0 ± 157.3	127.1 ± 147.4	0.019
Anti-MCV (U/ml)	122.7 ± 266.0	232.3 ± 447.6	401.0 ± 421.9	< 0.001
RF	51 (45.9%)	45 (47.9%)	71 (52.2%)	0.600
**Medications**				
Prednisolone	87 (78.4%)	92 (95.8%)	138 (100.0%)	< 0.001
Methotrexate	88 (79.3%)	82 (85.4%)	131 (94.9%)	0.001
Hydroxychloroquine	45 (40.5%)	58 (60.4%)	104 (75.4%)	< 0.001
Leflunomide	4 (3.6%)	30 (31.3%)	55 (39.9%)	< 0.001
Sulfasalazine	16 (14.4%)	21 (21.9%)	52 (37.7%)	< 0.001
Etanercept	2 (1.8%)	1 (1.0%)	3 (2.2%)	0.807
Adalimumab	0 (0.0%)	4 (4.2%)	6 (4.3%)	0.087
**Medication dosage**				
Prednisolone (mg/d)	3.8 ± 2.9	7.5 ± 3.5	9.2 ± 2.9	< 0.001
Methotrexate (mg/w)	7.9 ± 5.8	11.8 ± 7.1	15.3 ± 6.4	< 0.001
Hydroxychloroquine (mg/d)	81.1 ± 101.4	135.5 ± 124.7	184.8 ± 129.5	< 0.001
Leflunomide (mg/d)	0.7 ± 3.7	6.2 ± 9.3	7.8 ± 9.8	< 0.001
Sulfasalazine (g/d)	0.2 ± 0.4	0.4 ± 0.8	0.7 ± 1.0	< 0.001
Etanercept (mg/w)	0.4 ± 3.3	0.3 ± 2.5	0.7 ± 5.2	0.805
Adalimumab (mg/2w)	0.0 ± 0.0	1.5 ± 7.2	1.2 ± 5.8	0.087

RA: rheumatoid arthritis, VAS: Visual Analogue Scale, DAS-28-ESR: disease activity score 28 for RA with ESR, ESR: erythrocyte sedimentation rate, CRP: C-reactive protein, TSH: thyroid-stimulating hormone, FT3: free triiodothyronine, FT4: free thyroxin, anti-TPO: anti-thyroid peroxidase, anti-CCP: anti-cyclic-citrullinated peptide antibodies, anti-MCV: anti-mutated-citrullinated vimentin autoantibodies, RF: rheumatoid factor, SD: standard deviation. The P-value was significant if < 0.05

**Table 2:** Ordinal regression analysis determining independent predictors affecting rheumatoid arthritis disease severity

Variables	Adjusted OR	P-value
Age	1.008	0.370
Gender (female sex)	1.820	0.025
Duration of thyroid disease (year)	0.977	0.198
TSH (mIU/L)	1.117	0.027
FT3 (pg/mL)	1.696	< 0.001
FT4 (ng/dl)	1.153	0.011
Anti-TPO (IU/ml)	1.002	0.046

TSH: thyroid-stimulating hormone, FT3: free triiodothyronine, FT4: free thyroxin, anti-TPO: anti-thyroid peroxidase. The P-value < 0.05 was significant if < 0.05

## Discussion

The coexistence of RA with AITD has been long debated due to similar pathogenesis and the possible effect of RA treatment regiment on supression of thyroid hormones. However, a consensus on this matter is not yet achieved [12, 13]. Some studies have shown that Arthropathies and connective tissue diseases such as, RA are the most common autoimmuine disease coexisting with AITD [14, 15]. Moreover, several studies have established a higher prevalence of thyroid dysfunction, particularly overt hypothyroidism, anti-TPO positivity, and AITD among RA patients [16, 17]. In addition, recent studies have also suggested that AITD comorbidity can exacerbate RA severity and activity [18]. On the contrary, Joshi et al. found high RA disease activity was more common among patients with hypothyroidism, though it was not statistically significant [19]. Also, some studies have found no association between thyroid disease and RA disease activity which is in contrast with the findings of the aforementioned studies [20].

Due to conflicting results on this matter, we aimed to assess the association between RA severity and thyroid dysfunction, anti-TPO positivity, and AITD. In our study, 345 RA patients were included and were divided into three subgroups based on disease severity which was determined by the DAS-28-ESR score using. In each group, the clinical and paraclinical factors determining the severity of RA were analyzed regarding serum levels of thyroid function tests and thyroid autoantibodies. Our study’s data analysis yielded a significant association between RA disease severity and thyroid dysfunction, AITD, and anti-TPO positivity. Higher serum levels of TSH, FT3, FT4, and anti-TPO and the female gender could also influence the severity of RA disease.

Recent studies have shown that RA patients with hypothyroidism or positive anti-TPO had higher DAS-28-ESR levels [21].In addition, Koszarny et al., found a positive correlation between DAS-28-ESR and anti-thyroid antibodies, including anti-TPO levels [22]. However, Atzeni et al. found no correlation between anti-thyroid antibodies and RA disease activity parameters [23]. In our study, DAS-28-ESR was positively correlated with FT3 and anti-TPO serum levels.

Elattar et al. demonstrated that TSH level was positively correlated with other RA disease activity parameters, including Methotrexate dosage [18]. Although in our study, no correlation was found between the dosage of Methotrexate and TSH serum level, but positive correlation between Methotrexate and Hydroxychloroquine dosage and FT3 serum level were found. Consumption dosage of other medications such as Prednisolone was also positively correlated with FT3 and anti-TPO serum levels, but no significant correlation with other thyroid function tests was found. The aforementioned results demonstrate that FT3 may be a better measure of RA severity among the thyroid function tests.

Noteworthy, our study revealed that the patient’s age had no significant impact on disease activity which may have been due to the insufficient sample size. Another shortcoming of this study which leaves room for further studies investigations, is that thyroid ultrasound was only requested by the endocrinologist in patients with a suspicious history or physical examination findings in this study due to limited availability. In addition, other thyroid autoantibodies such as anti-thyrgobulin antibody were not measured due to the expensive costs and lack of availability, but it is noteworthy that the rate of isolated anti-thyroglobulin antibody positivity (presence of anti-thyroglobulin antibody without the presence of anti-TPO) seems to be minute [24, 25]; therefore, the impact of this matter on the eventual conclusion of the study seems to be insignificant. In conclusion, thyroid abnormality, including thyroid dysfunction and AITD comorbidity with RA disease, had a considerable impact on the RA disease severity, which can affect the quality of life and prognosis of the disease and widens the scope of approaching RA management and its numerous complications.

## Data Availability

The datasets generated and analyzed during the current study are not publicly available due to restrictions in ethical and data management approvals. Still, they are available from the corresponding authors upon reasonable request.

## List of abbreviations

RA: Rheumatoid arthritis
AITD: autoimmune thyroid disease
anti-TPO: anti-thyroid peroxidase
ACR/EULAR: American College of Rheumatology and the European League against Rheumatism
RF: rheumatoid factor
ESR: erythrocyte sedimentation rate
CRP: C-reactive protein
anti-MCV: anti-mutated-citrullinated vimentin autoantibodies
anti-CCP: anti-cyclic-citrullinated peptide antibodies
ELISA: enzyme-linked immunosorbent assay
RF: rheumatoid factor
CLIA: chemiluminescent immunoassay
TSH: thyroid stimulating hormone
FT4: free thyroxin
FT3: free triiodothyronine
DAS-28-ESR: disease activity score in 28 Joints for RA with ESR
VAS: visual analog scale
SD: standard deviation
OR: odds ratio
CI: confidence interval
